# Rapid phosphatidic acid accumulation in response to low temperature stress in *Arabidopsis* is generated through diacylglycerol kinase

**DOI:** 10.3389/fpls.2013.00001

**Published:** 2013-01-22

**Authors:** Steven A. Arisz, Ringo van Wijk, Wendy Roels, Jian-Kang Zhu, Michel A. Haring, Teun Munnik

**Affiliations:** ^1^Department of Plant Physiology, Swammerdam Institute for Life Sciences, University of AmsterdamAmsterdam, Netherlands; ^2^Department of Horticulture and Landscape Architecture, Purdue UniversityWest Lafayette, IN, USA; ^3^Shanghai Center for Plant Stress Biology and Shanghai Institute of Plant Physiology and Ecology, Shanghai Institutes of Biological Sciences, Chinese Academy of SciencesShanghai, China

**Keywords:** abiotic stress, cold stress, diacylglycerol kinase, lipid signaling, phosphatidic acid, phosphoinositide, phospholipase, plant signaling

## Abstract

Phosphatidic acid (PtdOH) is emerging as an important signaling lipid in abiotic stress responses in plants. The effect of cold stress was monitored using ^32^P-labeled seedlings and leaf discs of *Arabidopsis thaliana*. Low, non-freezing temperatures were found to trigger a very rapid ^32^P-PtdOH increase, peaking within 2 and 5 min, respectively. In principle, PtdOH can be generated through three different pathways, i.e., (1) via *de novo* phospholipid biosynthesis (through acylation of lyso-PtdOH), (2) via phospholipase D hydrolysis of structural phospholipids, or (3) via phosphorylation of diacylglycerol (DAG) by DAG kinase (DGK). Using a differential ^32^P-labeling protocol and a PLD-transphosphatidylation assay, evidence is provided that the rapid ^32^P-PtdOH response was primarily generated through DGK. A simultaneous decrease in the levels of ^32^P-PtdInsP, correlating in time, temperature dependency, and magnitude with the increase in ^32^P-PtdOH, suggested that a PtdInsP-hydrolyzing PLC generated the DAG in this reaction. Testing T-DNA insertion lines available for the seven *DGK* genes, revealed no clear changes in ^32^P-PtdOH responses, suggesting functional redundancy. Similarly, known cold-stress mutants were analyzed to investigate whether the PtdOH response acted downstream of the respective gene products. The *hos1, los1*, and *fry1* mutants were found to exhibit normal PtdOH responses. Slight changes were found for *ice1, snow1*, and the overexpression line *Super-ICE1*, however, this was not cold-specific and likely due to pleiotropic effects. A tentative model illustrating direct cold effects on phospholipid metabolism is presented.

## Introduction

The potential to survive low temperatures is one of the factors that determine the geographical distribution of plants. Moreover, freezing and cold stress restrict the arable land and yield of crops. Therefore, much effort is made to understand the mechanisms that make plants more tolerant to low temperatures. One of the most popular plant models in these studies is *Arabidopsis thaliana* (Van Buskirk and Thomashow, 2006).

Like many temperate plants, *Arabidopsis* is capable of cold acclimation, i.e., during a period of cold, non-freezing temperatures, its tolerance for freezing temperatures increases. This process, also referred to as cold hardening, involves a myriad of metabolic and developmental changes, accompanied by accumulation of proteins and compatible solutes, and alterations in membrane composition (Uemura et al., 1995; Thomashow, 1999; Cook et al., 2004; Chinnusamy et al., 2007).

Transcriptome profiling and mutant screens have resulted in the characterization of multiple genes involved in the initiation of cold acclimation and freezing tolerance. These include the conserved CBF/DREB1 transcription factors that are responsible for activating the expression of many cold response (COR) genes via conserved C-repeat elements in their promoters. Zhu and coworkers have used *Arabidopsis* plants transfected with the *RD29A-LUC* construct to select for mutants with altered responses to cold treatment (“cold response mutants”), resulting in the identification of several genes. Enhanced cold-induced expression was found in the *fry1* (Xiong et al., 2001) and *hos1* (Ishitani et al., 1998) mutants, whereas *los1* (Guo et al., 2002) showed decreased expression. Moreover, the dominant negative *ice1* (Chinnusamy et al., 2003) mutation has been demonstrated to negatively affect cold-induced gene transcription by interfering with the function of *AtICE1*, a *myc*-type transcription factor which functions in CBF transcription in cold signaling. The *myb*-type transcription factor SNOW1/MYB15, also binds to the CBF promoter region, interacting with ICE1 (Agarwal et al., 2006). Upon exposure to cold stress (4°C), the transcript levels of CBF/DREB1 genes increase within 15–30 min, followed by the accumulation of COR gene transcripts after about 2 h (Thomashow, 1999).

Much less is known about the signal transduction pathway that preceeds the gene expression changes. Nonetheless, there is mounting evidence that Ca^2+^ functions as a second messenger (Knight et al., 1996; Knight and Knight, 2000; Carpaneto et al., 2007) and that part of the pathway involves activation of a MAP kinase cascade (Jonak et al., 1996; Mizoguchi et al., 1996; Teige et al., 2004). One of the latest additions to the field of cold signaling is the formation of the lipid second messenger, phosphatidic acid (PtdOH). In suspension-cultured cells, this phospholipid was shown to accumulate within minutes of cold stress (Gawer et al., 1999; Ruelland et al., 2002; Cantrel et al., 2011). Like Ca^2+^ and MAP kinases, PtdOH is involved in the signal transduction pathways of several other plant stress responses, including drought, wounding, and pathogen infection (Li et al., 2009; Testerink and Munnik, 2011), and it is not unlikely that these pathways strongly overlap.

In stress-induced signal transduction, PtdOH responses have been mainly attributed to two pathways. It is the direct product of phospholipase D (PLD), which hydrolyses structural phosholipids like phosphatidylcholine (PtdCho) and phosphatidylethanolamine (PtdEtn), and a secondary product of the phospholipase C (PLC) pathway, which first hydrolyzes polyphosphoinositides (PPIs) to diacylglycerol (DAG), that is subsequently phosphorylated to PtdOH by diacylglycerol kinase (DGK). However, metabolism of DAG and PtdOH is more complex, since multiple sources have now been demonstrated, and PtdOH is formed *de novo* via acylation of glycerolphosphate (Gro3P) as a common intermediate in glycerolipid biosynthesis, both in the plastid and the ER. Thus, PtdOH is precursor to all phosphoglycerolipids as well as triacylglycerols and galactolipids, and its turnover is crucial in determining lipid metabolic fluxes and membrane compositions.

The *Arabidopsis* genome is predicted to encode 12 PLDs, 9 PLCs, and 7 DGKs (Gomez-Merino et al., 2004; Testerink and Munnik, 2005, 2011; Tasma et al., 2008; Arisz et al., 2009; Li et al., 2009; Munnik and Testerink, 2009). Their genetic abundance and specific gene expression patterns suggests that some of these enzymes are specific to certain locations in specific organs and/or involved in distinct processes. The PLC/DGK and PLD pathways have been implicated in the transcriptional induction of an array of cold-induced genes in *Arabidopsis* (Vergnolle et al., 2005). PLDδ has been shown to be important in the generation of freezing tolerance during acclimation (Li et al., 2004) in contrast to PLDα1 which negatively influenced survival of freezing, both in cold-acclimated and in non-acclimated plants (Rajashekar et al., 2006; Chen et al., 2008; Du et al., 2010). In suspension-cultured cells, biochemical evidence was found that cold shock activated both PLC/DGK and PLD pathways (Ruelland et al., 2002). Moreover, several genes have been shown to be upregulated in response to cold stress, including *PLDα1, PLDδ, PLC1, PLC4, PLC5, DGK1*, and *DGK2* (Hirayama et al., 1995; Gomez-Merino et al., 2004; Li et al., 2004; Lee et al., 2005). Also, in maize roots and leaves several genes encoding DGK, PLC, and PLD were upregulated within 30 min of cold stress (Sui et al., 2008).

In this study we show that *Arabidopsis* seedlings and leaf disks exposed to low temperatures accumulate PtdOH within minutes. Using a differential ^32^P-labeling strategy (Munnik et al., 1998b; Arisz et al., 2009) and PLD's ability to transphosphatidylate n-butanol to PtdBut (Munnik et al., 1995, 1998b), we provide evidence that the rapid PtdOH response does not originate from PLD but from DGK. The simultaneous decrease in the level of phosphatidylinositolphosphate (PtdInsP) suggests the involvement of a PtdInsP-hydrolyzing PLC. T-DNA insertion lines were used to address the question which DGK and PLC were involved, while the COR mutants *hos1, los1, fry1, ice1*, and *snow1* were analyzed to see whether PtdOH acts up- or down-stream of these genes in the COR.

## Materials and methods

### Plant material

*A. thaliana* seeds were sterilized in 70% EtOH (1 min) and 25% bleach (20 min), and sown on media in Petri dishes. For ^32^P-radiolabeling experiments, seedlings were grown on ½ x Murashige and Skoog (MS) basal medium at pH 5.7 (KOH), solidified with 1.0 % bacto-agar. The *ice1, snow1, los1, hos1, fry1* mutants, and their WT's were grown on 1 x MS medium supplemented with 1% sucrose. A 16 h light/8 h dark regime (150 umol quanta m^−2^s^−1^) at 21°C was set. To promote uniform germination, plates were kept in the dark at 4°C for 2 days before transfer to a climate room.

### RT-PCR expression analyses of DGK T-DNA insertion lines

Homozygous T-DNA insertion lines of the *DGK* genes where genotyped using primer sequences found in Table A1 (Figure A3). Wild type *A. thaliana Col-0* or lines containing T-DNA insertions in *DGK1, -3, -5, -7* genes were grown on ½ x Murashige and Skoog (MS) basal medium supplemented with 1% w/v sucrose at pH 4.6, solidified with 1% w/v daishin agar. To promote uniform germination, plates were kept in the dark at 4°C for 2 days before transfer to a climate room. Seedlings where harvested for RNA isolation after 9 days in a climate room with light regime set at 16 h light/8 h dark at 21°C and Relative Humidity 70%. Additionally, flowers of *A. thaliana* lines containing T-DNA insertions in *DGK2, -4, -6* genes, and wild type *Col-0* were collected from plants grown in a greenhouse under the same environmental conditions. RNA was isolated using Tri Reagens LS (Sigma) and treated with Turbo RNAse free DNAse (Ambion) for removal of genomic DNA. The RNA concentration and integrity was analyzed using a Nanodrop ND-1000 spectrophotometer.

cDNA was synthesized from 2 μg total RNA using RevertAid H Minus Reverse Transcriptase (Fermentas) according to the manufacturers protocol. RT-PCR was performed using Accuprime Taq DNA polymerase (Invitrogen). Table A2 contains the primer sequences used to amplify the different *Arabidopsis DGK* genes and the *At2g28390* (SAND family) reference gene (Figure A4; Hong et al., 2010). Thermal cycling was done according to the following profile; 94°C for 2 min, followed by 40 cycles of 94°C for 30 s, 50°C for 30 s, 68°C for 2 min and 1 cycle of 68°C for 6 min.

### ^32^P-orthophosphate radiolabeling *in vivo* and analysis of phospholipids

Five-days-old seedlings or leaf disks (5 mm ∅) of 3-weeks-old plants were transferred to a 2.0 ml Eppendorf tube, containing MES (2-[N-morpholino]ethane sulfonic acid)-based buffer of 2.56 mM MES (pH 5.7) and 1 mM KCl. To label phospholipids, 10 μCi carrier-free ^32^P-orthophosphate per tube was added for 16 h, unless indicated otherwise. Cold shock treatments were executed by transferring tubes to ice water. Incubations were stopped by the addition of HClO_4_ (final concentration 5%, w/v), and 10 min of subsequent shaking.

The total solvent was removed and 375 μl CHCl_3_/MeOH/HCl (50:100:1, by vol.) was added to extract the lipids. After 10 min of vigourous shaking, two phases were induced by adding 375 μl CHCl_3_ and 200 μl 0.9% (w/v) NaCl. The organic lower phase was then transferred to a tube containing 375 μl CHCl_3_/MeOH/1M HCl (3:48:47, by vol.). Shaking, spinning, and removing the upper phase yielded a purified organic phase, which was dried down in a vacuum centrifuge at 50°C. The residue was resuspended in 50 μl CHCl_3_ and sampled for lipid analysis.

Phospholipids were analyzed by thin-layer chromatography (TLC) on heat-activated silica gel 60 plates (Merck, 20 × 20 cm) using one of the following solvent systems (ratios by vol.): (A) CHCl_3_/MeOH/NH_4_OH (25%)/H_2_O (90:70:4:16); or (B) ethylacetate/*iso*-octane/formic acid/H_2_O (13:2:3:10), of which the organic phase was used for TLC. Solvent A was used for total phospholipid analysis, while B was used to quantitate PtdOH and PtdBut. Radiolabeled phospholipids were visualized and quantified by phosphoimaging (Molecular Dynamics, Sunnyvale CA, USA).

## Results

### Cold stress rapidly triggers a PtdOH response

**Figure 1:**
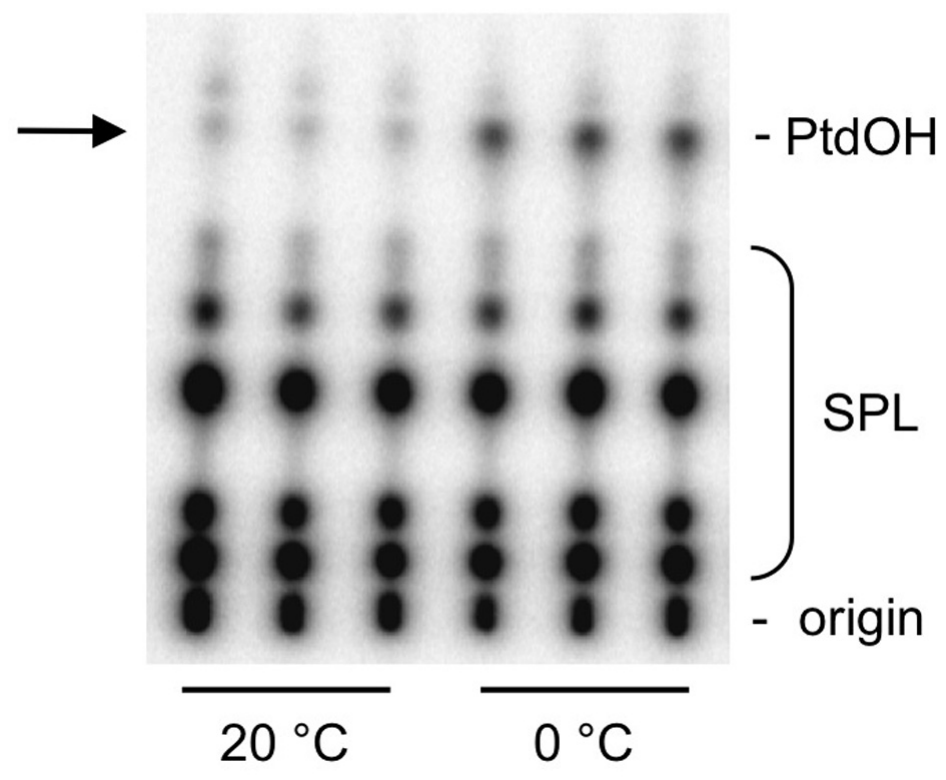
**Cold stress triggers the formation of ^32^P-PtdOH in *Arabidopsis* seedlings.** Five-days-old seedlings were metabolically radiolabeled O/N with ^32^P_i_ and then incubated for 5 min at 0°C or maintained at 20°C. Lipids were extracted, separated by TLC, and visualized by phosphoimaging. Each lane represents an extract of two seedlings. *Abbreviation*: SPL, structural phospholipids.

PtdOH levels in plants are approximately 2 mol% of total phospholipids (Welti et al., 2002) which likely represents ER- and plastid-localized PtdOH as precursor and turnover product of structural glycerolipids. To be able to see PtdOH increases during stress-signaling, plants can be metabolically radiolabeled with carrier-free ^32^P-phosphate (^32^P_i_). To study phospholipid metabolism during cold shock in *Arabidopsis*, we radiolabeled 5-days-old seedlings for 16 h with ^32^P_i_ and subsequently incubated them for 5 min at 0°C. Phospholipids were then extracted, separated by TLC and analyzed by autoradiography. A typical ^32^P-labeling pattern is shown in Figure 1, revealing a PtdOH increase in response to cold.

**Figure 2:**
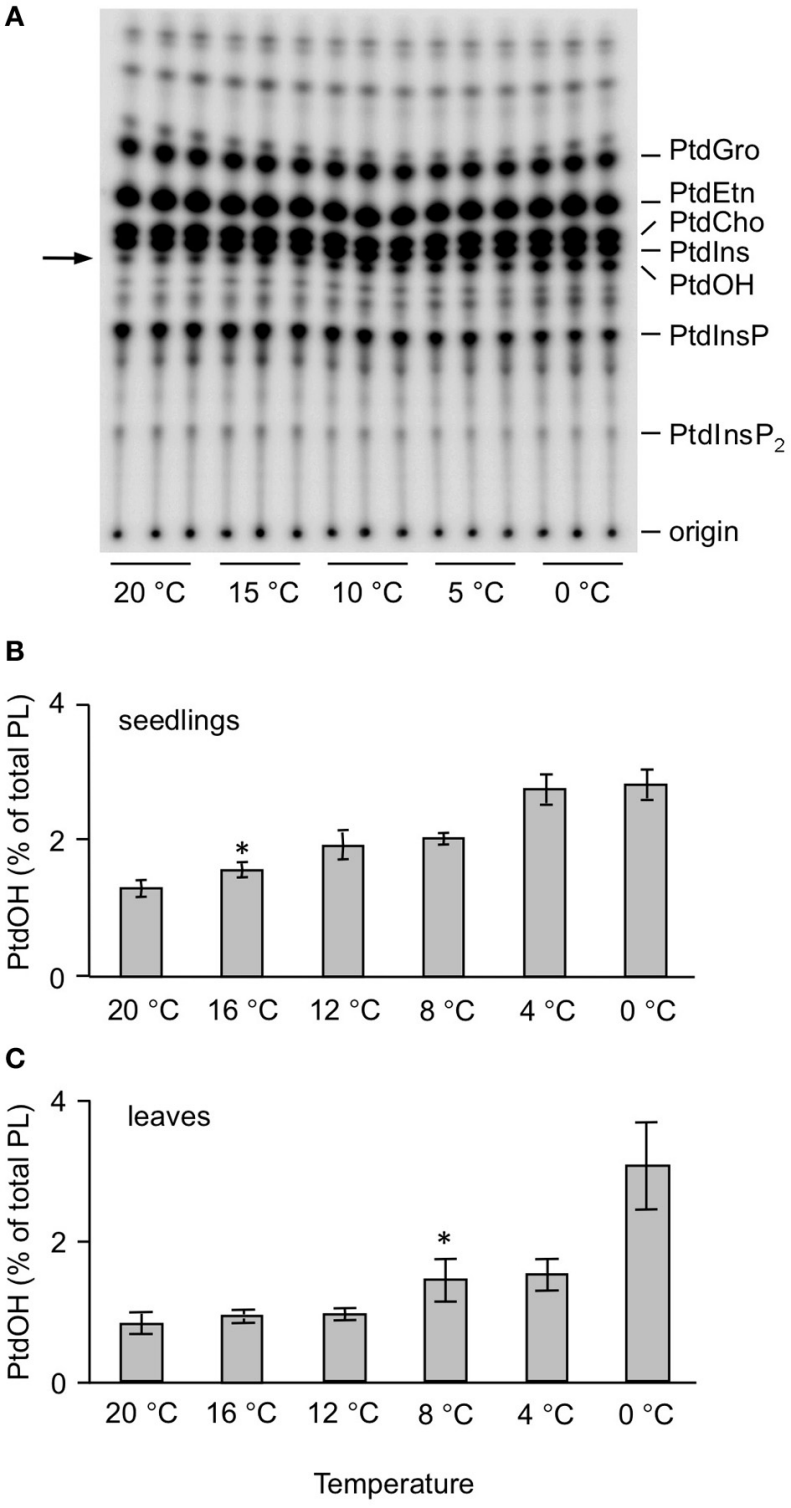
**Temperature-dependent accumulation of ^32^P-PtdOH in *Arabidopsis* leaves and seedlings. (A)** O/N ^32^P-prelabeled seedlings were incubated for 5 min at the indicated temperatures. Lipids were then extracted, separated by TLC, and visualized by autoradiography. **(B)** Quantitation by phosphoimaging of ^32^P-PtdOH formed at different temperatures in seedlings. **(C)** Formation of ^32^P-PtdOH in leaf disks at different temperatures. Values are means of triplicates ±SD. Asterisks indicate highest temperatures giving rise to a significant (*p* < 0.05) increase in ^32^P-PtdOH.

To test the temperature dependency of this response, ^32^P_i_-prelabeled seedlings were exposed to different temperatures for 5 min. As shown in Figures 2A,B, a temperature-dependent PtdOH response was found. Concomitantly, a decrease in ^32^P-PtdInsP was observed (Figure 2A). To investigate whether leaves of adult plants responded similarly, leaf disks of 3-weeks-old plants were subjected to the same labeling procedure and temperature treatments. Quantitation of the PtdOH levels by phosphoimaging revealed a significant response at 8°C or lower (Figure 2C), which is different for seedlings which already responded to a shift to 16°C (Figure 2B).

**Figure 3:**
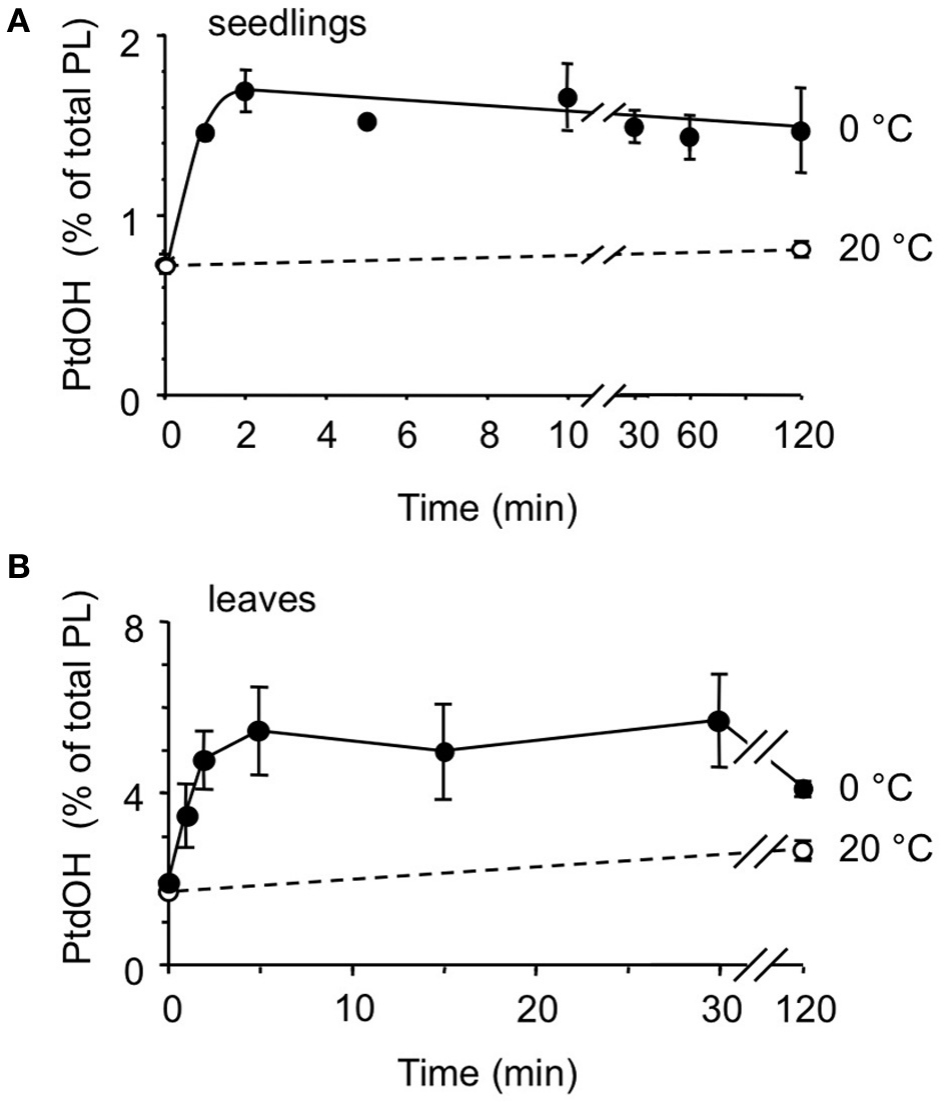
**Kinetics of cold-induced ^32^P-PtdOH accumulation in *Arabidopsis* seedlings and leaves.** ^32^P-prelabeled seedlings **(A)** or leaf disks **(B)** were incubated at 0°C (filled circles) or 20°C (control, open circles) for different periods of time. Lipids were then extracted, separated by TLC, and quantified by phosphoimaging. Data points (±SD) are from triplicate incubations.

Next, the kinetics of the PtdOH response was investigated. As shown in Figure 3A, PtdOH accumulation at 0°C in seedlings reached a maximum within 2 min and then leveled off, staying up for at least 2 h. The response of leaf disks of adult plants to 0°C was found to be slightly slower but was still relatively fast, peaking at 5 min after the onset of incubation after which it leveled off, approaching control levels after 2 h (Figure 3B).

### The rapid cold induced-PtdOH response is generated by DGK, not by PLD activity

Next, we focused on the metabolic origin of the cold-induced PtdOH response. Previous studies in suspension-cultured *Arabidopsis* cells indicated that part of the cold shock-induced PtdOH response was generated by PLD activity (Ruelland et al., 2002). To investigate PLD's contribution a transphosphatidylation assay was performed, i.e., in the presence of a low concentration of a primary alcohol, such as n-ButOH, this serves as a substrate in a PLD-catalyzed reaction generating PtdBut, at the cost of PLD-catalyzed production of PtdOH (Munnik et al., 1995). The accumulation of PtdBut is a measure of PLD activity.

**Figure 4:**
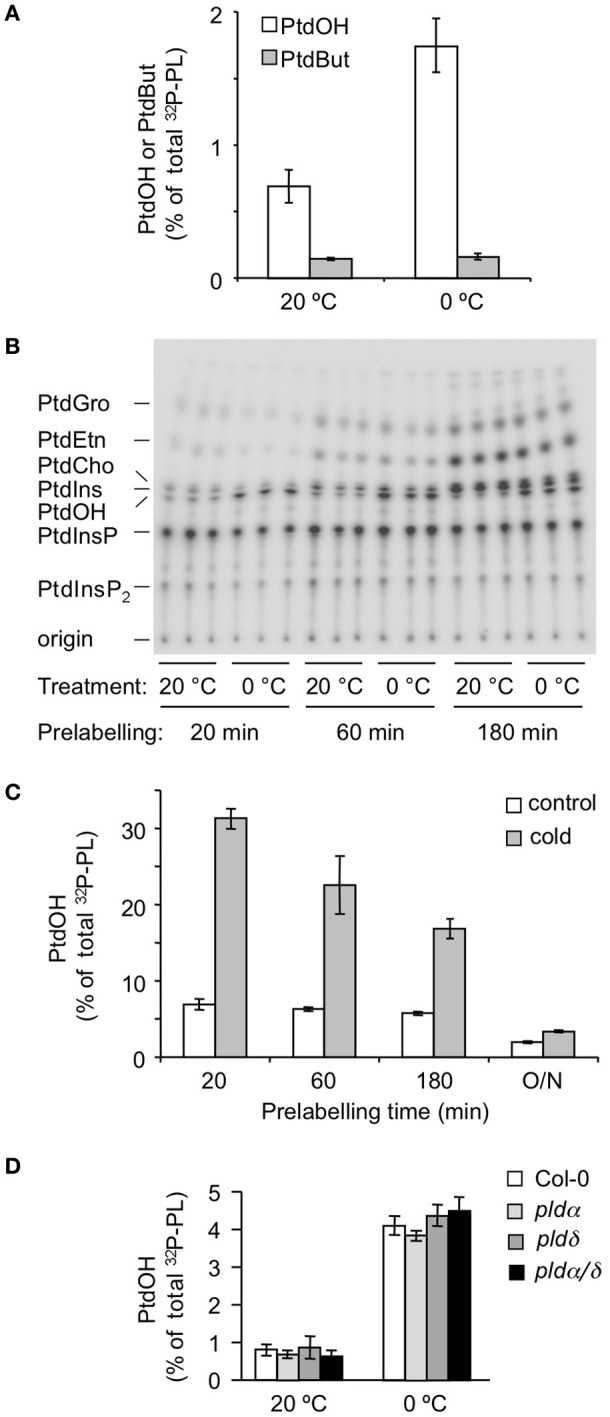
**Metabolic origin of the chilling-induced ^32^P-PtdOH response in *Arabidopsis* seedlings. (A)** In the presence of 0.5% n-butanol, accumulation of the transphosphatidylation product ^32^P-PtdBut is used as measure of PLD activity. White bars, ^32^P-PtdOH, gray bars, ^32^P-PtdBut. **(B)** Seedlings were prelabeled with ^32^P_i_ for 20, 60, or 180 min, to preferentially label the monoester-phosphates of lipids with high turnover rates. Subsequently, seedlings were transferred to cold (0°C) or kept at 20°C for an additional 15 min. Lipids were separated on TLC and visualized by phosphoimaging. **(C)** Dependence of ^32^P-PtdOH levels in control (white bars) and cold conditions (gray bars) on the ^32^P-prelabeling time. **(D)** Five-days old Col-0, *plda1, pldd*, and *plda1/d* knock-out seedlings were radiolabeled O/N with ^32^P_i_ and then incubated for 5 min at 0°C or maintained at 20°C. ^32^P-PtdOH increases are expressed as percentage of total ^32^P-labeled lipids.

Thus, seedlings were prelabeled for 16 h with ^32^P_i_, then n-ButOH (0.5% final conc.) was added, and 30 min later the seedlings were transferred to 0°C for 5 min or kept at room temperature. As shown in Figure 4A, cold stress did not affect ^32^P-PtdBut levels, while ^32^P-PtdOH levels increased. These data indicate that PLD is not responsible for the initial PtdOH response.

To investigate the potential involvement of DGK, a differential radiolabeling protocol was applied (Munnik, 2001; Arisz et al., 2009). In short, when cells are metabolically labeled with ^32^P_i_, the phospholipid classes are labeled with different kinetics, depending on the labeling of their precursors, their rates of synthesis, turnover, and pool size. Thus, DGK-derived PtdOH is labeled after relatively short labeling times because it acquires its ^32^P-phosphate directly from ATP molecules, which are rapidly labeled. This is in contrast to PtdOH arising from PLD activity, which are not labeled until the pool of its precursor, i.e., PtdEtn, PtdCho, or PtdGro, is sufficiently labeled, which is typically O/N (Munnik et al., 1998b; Arisz and Munnik, 2011).

Thus, seedlings were ^32^P_i_-prelabeled for different periods of time (20, 60, and 180 min) after which they were subjected for 5 min to 0°C. As shown in Figures 4B,C, cold stress triggered a marked increase in ^32^P-PtdOH in seedlings prelabeled for only 20 min. Under these conditions, structural phospholipids like PC and PE were hardly labeled excluding them as precursors to ^32^P-PtdOH in a PLD-catalyzed reaction. This is in agreement with the results of the transphosphatidylation assay (Figure 4A). Hence, the increase in ^32^P-PtdOH is unlikely to reflect a PLD activity, and is consistent with a DGK activity. At longer prelabeling time points, the relative increases in ^32^P-PtdOH gradually diminished (Figure 4C), due to the decrease in the specific radioactivity of the ATP pool and the general increase in structural phospholipid labeling.

Two of the most abundant PLD isoforms in Arabidopsis, *PLDα1* and *PLDδ*, have been implicated in cold stress tolerance (Ruelland et al., 2002; Li et al., 2004; Rajashekar et al., 2006). To test their contribution to the early PtdOH response to cold stress, both single and double KO-mutants were analyzed (Bargmann et al., 2009a,b). As shown in Figure 4D, all mutants exhibited a normal PtdOH response upon cold stress.

Together, these results argue against the involvement of PLD in the acute cold-shock-induced PtdOH response and strongly point to a role for DGK.

**Figure 5:**
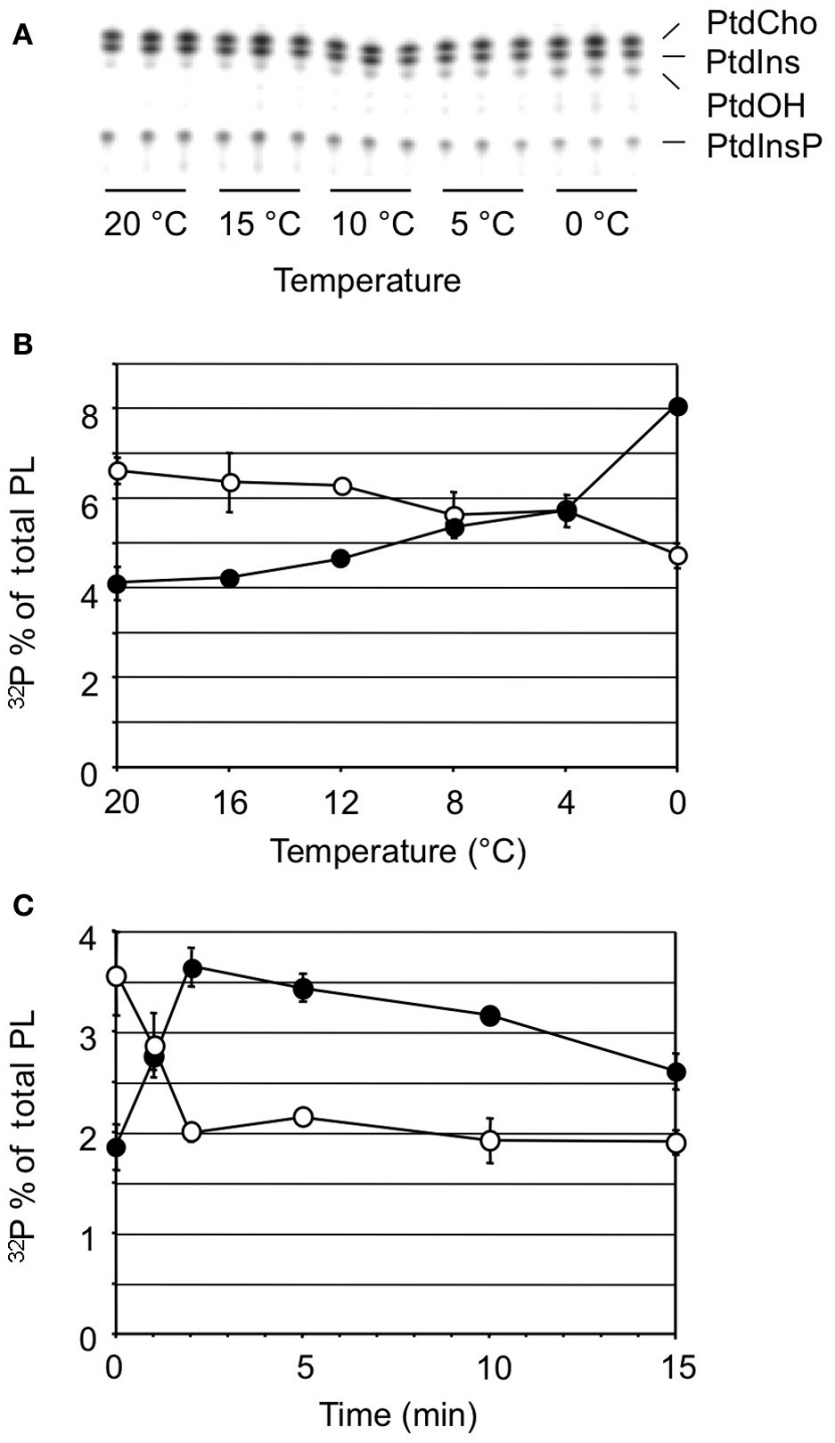
**Cold stress-induced changes in ^32^P-PtdOH vs. ^32^P-PtdInsP. (A)** TLC analysis of ^32^P-phospholipids extracted from seedlings after 5 min exposure to the temperatures indicated. **(B)** Similar experiment as **(A)** Quantitation of radioactivity in the lipids was by phosphoimaging. Filled circles, ^32^P-PtdOH; open circles, ^32^P-PtdInsP. **(C)** A time course experiment at 0°C shows contrary changes in ^32^P-PtdOH and ^32^P-PtdInsP. All values are means of at least three samples containing two seedlings each from a representative experiment (error bars indicate SD).

The implication of DGK in the early COR raised the question of DAG's metabolic origin. One possible source of DAG is the induced PLC hydrolysis of the polyphosphoinositides, PtdInsP and/or PtdInsP_2_, a well-defined plant stress response, which was supported by the observation that ^32^P-PtdInsP decreased in response to cold (Figure 2A). Moreover, this decrease correlated closely with an equivalent increase in ^32^P-PtdOH, in a temperature- and time-dependent fashion (Figure 5). These results strongly argue for the scenario that cold stress activates PLC hydrolysis of PtdInsP to form DAG, which is subsequently phosphorylated to PtdOH by DGK.

### PtdOH responses in arabidopsis dgk- and cold stress mutants

*Arabidopsis* contains 7 DGK encoding genes. In an attempt to identify the isozyme involved in the cold-induced PtdOH response, a reversed genetic approach was used, screening a series of T-DNA insertion lines (Tables 1, 2). These lines carry insertions in or near the *DGK* encoding regions, although not all of the lines were established as knockout or knockdown mutants (Table 1; Figure A4). Seedlings of these lines were ^32^P_i_-prelabeled (O/N) and then exposed to 0°C for 5 min to measure their PtdOH response. Surprisingly, among the lines with normal control PtdOH levels, neither showed significant defects in the cold-induction of PtdOH, whilst slight larger PtdOH increases were found in cold-stressed *dgk7-2* seedlings (Table 1). In leaf discs, *dgk6-1, dgk7-1*, and *dgk7-2* revealed slightly enhanced levels of cold-induced PtdOH (Table 2). Clearly, redundancy is involved and some of the KO/KD lines may constitutively upregulate genes that even mask or enhance the response.

**Table 1:** **Cold-induced PtdOH formation in seedlings of T-DNA insertion lines**.

**Gene**	**AGI ID**	**Line**	**Name**	Status^*^	**Control**	**Cold**	**Fold increase**	**Replicates**
		wt	*Col-0*	wt	0.98 ± 0.07	3.00 ± 0.20	3.1	6
*AtDGK1*	At5g07920	SALK 053412	*dgk1-1*	KD	0.93 ± 0.07	3.11 ± 0.31	3.4	6
*AtDGK2*	At5g63770	SAIL 718_G03	*dgk2-1*	KO	1.00 ± 0.06	2.70 ± 0.48	2.7	6
		SAIL 71_B03	*dgk2-2*	KO	0.96 ± 0.10	3.19 ± 0.40	3.3	6
*AtDGK4*	At2g20900	SAIL 339_C01	*dgk4-1*	no KO	1.22 ± 0.08	3.73 ± 0.26	3.1	3
		SALK 069158	*dgk4-2*	KO	0.83 ± 0.08	3.65 ± 0.50	4.4	3
*AtDGK5*	At2g20900	SAIL 1212_E10	*dgk5-1*	KO	0.85 ± 0.07	3.73 ± 0.15	4.4	3
*AtDGK6*	At4g28130	SALK 016285	*dgk6-1*	ND	1.08 ± 0.03	4.48 ± 0.55	4.1	3
*AtDGK7*	At4g30340	SAIL 51_E04	*dgk7-1*	KD	0.90 ± 0.17	2.92 ± 0.22	3.2	8
		SALK 059060	*dgk7-2*	KD	0.91 ± 0.14	3.24 ± 0.15^**^	3.6	8
		SALK 007896	*dgk7-3*	no KO	0.87 ± 0.14	2.94 ± 0.19	3.4	8

5-days-old Arabidopsis seedlings were labeled O/N with ^32^P_i_ and incubated at 0°C for 5 min. Lipids was then extracted separated by TLC and quantified by phosphoimaging. PtdOH levels are expressed as a percentage of the total ^32^P-labeled lipids and values represent averages of multiple samples containing 2 seedlings each (± SD).

* Transcript analysis by RT-PCR confirmed knock-down (KD) or knock-out (KO) status. Expression of DGK6 was too low for detection hence the status of dgk6-1 could not be determined.

** Reproducible statistically significant difference of T-DNA line compared with wild type (Tukey HSD test, P < 0.05) within the wild type control homogeneous subset.

**Table 2:** **Cold-induced PtdOH formation in leaf disks of T-DNA insertion lines**.

	**Control**	**Cold**	**Fold increase**
*Col-0*	2.1 , 0.7	6.0 , 1.1	2.9
*dgk1-1*	2.2 , 0.5	5.1 , 0.4	2.3
*dgk2-1*	2.2 , 0.6	6.7 , 1.7	3.1
*dgk2-2*	1.9 , 0.3	6.5 , 1.8	3.3
*dgk4-1*	1.9 , 0.4	5.9 , 0.9	3.2
*dgk4-2*	2.0 , 0.3	7.2 , 1.0	3.7
*dgk5-1*	3.3 , 0.3	8.4 , 0.4	2.6
*dgk6-1*	2.6 , 0.2	9.7 , 0.6^*^	3.7
*dgk7-1*	2.0 , 0.4	9.4 , 1.4^*^	4.6
*dgk7-2*	2.3 , 0.5	10.3 , 1.0^**^	4.5
*dgk7-3*	1.8 , 0.2	8.7 , 0.5	4.9

Rosette leaf disks of 5–6-weeks-old Arabidopsis plants were labeled O/N with ^32^P_i_ and incubated at 0°C for 5 min. Lipids were extracted, separated by TLC and quantified by phosphoimaging. PtdOH levels are expressed as percentage of the total ^32^P-labeled lipids. Values are averages ±SD (n ≥ 3).

^*,**^ Reproducible statistically significant difference of T-DNA line compared with wild type (Tukey HSD test,

* P < 0.05;

** P < 0.01) within the wild type control homogeneous subset.

**Figure 6:**
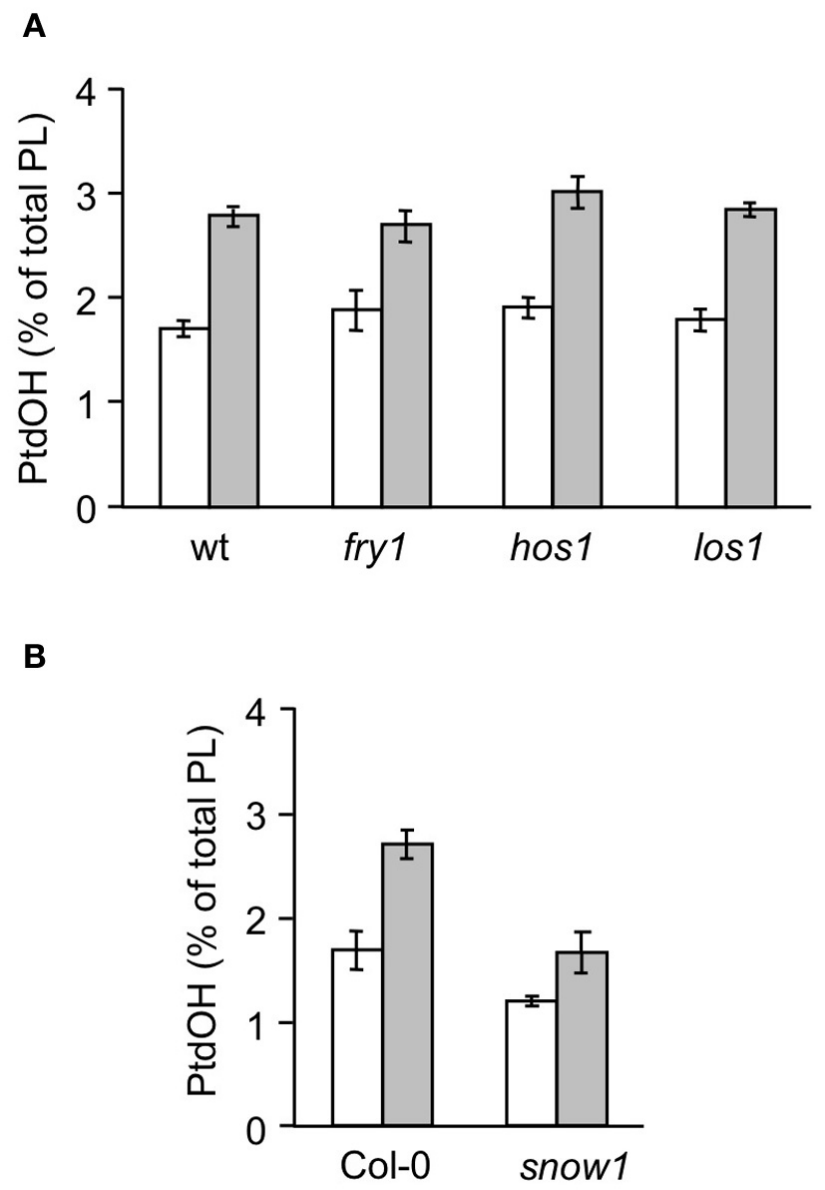
**Cold-induced ^32^P-PtdOH induction in known *Arabidopsis* cold response mutants.** Five-days-old seedlings were prelabeled O/N with ^32^P_i_ and subsequently incubated at 0°C or kept at room temperature for 15 min. Lipids were then extracted, separated by TLC, and quantified by phosphoimaging. ^32^P-PtdOH levels are expressed as percentage of the total ^32^P-lipid. Values are means from triplicate incubations from a typical experiment; error bars indicate SD. White bars, control; gray bars, 0°C. **(A)** The mutants *fry1, hos1, los1*, and their wt background, C24*RD29A-LUC*. **(B)** The *snow1* mutant and the wt control, Col-0.

**Figure 7:**
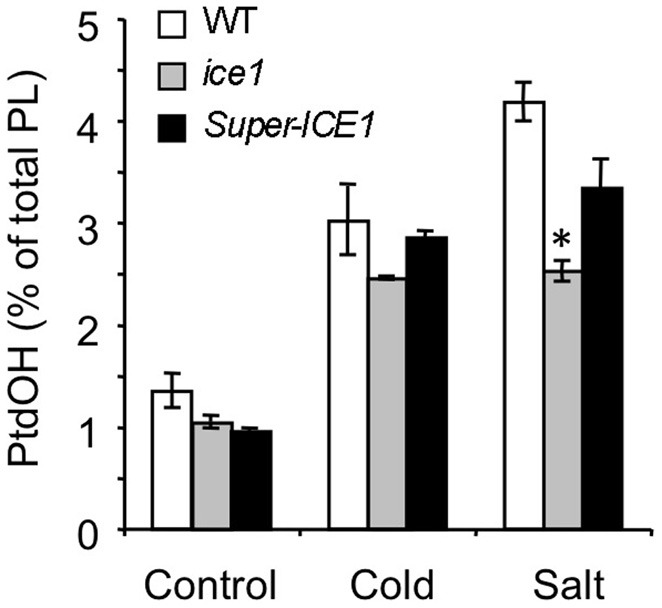
**^32^P-PtdOH responses in seedlings of the *ice1* mutant and *ICE1* overexpression transgenic line (*Super-ICE1*).** Five-days-old seedlings were prelabeled O/N with ^32^P_i_ and incubated at 0°C or with 300 mM NaCl for 15 min. ^32^P-PtdOH levels are expressed as percentage of the total ^32^P-lipid (average ±SD). ^32^P-PtdOH was enhanced due to cold and salt in all genotypes (*p* < 0.025), but salt-induced ^32^P-PtdOH was decreased in *ice1* compared to wildtype (*p = 0.008).

In Arabidopsis, several COR mutants have been identified, including *ice1, snow1, fry1, hos1*, and *los1* [(Ishitani et al., 1998; Xiong et al., 2001; Guo et al., 2002; Chinnusamy et al., 2003) mutation has been demonstrated to negatively affect cold-induced gene transcription by interfering with the function of *AtICE1*, a *myc*-type transcription factor which functions in CBF transcription in cold signaling. The *myb*-type transcription factor SNOW1/MYB15, also binds to the CBF promoter region, interacting with ICE1 (Agarwal et al., 2006)]. To gain information on the position of the PtdOH response in the cold sensing pathway, each mutant was analyzed for its cold-induced PtdOH response. As shown in Figure 6A, *fry1, hos1*, and *los1* all showed a normal response, but *snow1* had a lower basal and cold shock-induced level of ^32^P-PtdOH (*p* = 0.006; Figure 6B); nevertheless, the relative stimulation levels were not significantly altered. Although the cold-induced PtdOH response in *ice1* generally appeared to be lower than wildtype (Figure 7), it did not reach the significance level and was not cold-specific either, because the PtdOH response induced by salt stress (300 mM NaCl, 15 min) was also decreased (*p* = 0.008). Since these seedlings look stunted, pleiotropic effects are most likely to account for the observed differences. Similarly, PtdOH levels in the overexpressor of *ICE1, Super-ICE1*, tended to be suppressed, again indicating pleiotropic effects (Figure 7). Together these results indicate that the PtdOH response is upstream.

## Discussion

While, in *Arabidopsis* seedlings and leaves, the acclimation process in response to low temperatures is rapidly initiated, it takes 6–7 days to achieve maximal freezing tolerance (Uemura et al., 1995; Peng et al., 2007). The formation of PtdOH has been speculated to function in the regulation of this response (Ruelland et al., 2002; Xiong et al., 2002; Gomez-Merino et al., 2004; Li et al., 2004; Vergnolle et al., 2005; Rajashekar et al., 2006). While this has previously been studied in suspension-cultured cells, we have focused on the response in whole seedlings and mature leaves. The results showed that cold shock treatment triggered a rapid and sustained (during hours) accumulation of PtdOH, both in seedlings and in leaf discs of mature *Arabidopsis* plants (Figures 1–3). The leaf response was generally more pronounced, but in seedlings the PtdOH increase was faster and already visible upon minor temperature shifts which did not lead to a response in leaves. Since the accumulation of PtdOH is emerging as a common early element in environmental stress responses, and because it is suggested to be involved in the acclimation process, it is important to have knowledge of the underlying mechanisms.

### DGK rather than PLD activity generates early, cold-induced PtdOH

Two routes have been found to generate PtdOH under conditions of environmental stress in plants, i.e., PLD hydrolysis of strucural phospholipids (i.e., PtdCho/PtdEtn/PtdGro) and phosphorylation of DAG by DGK (Arisz et al., 2009; Testerink and Munnik, 2011). Using transphosphatidylation assays, the absence of a ^32^P-PtdBut increase under chilling conditions that triggered massive ^32^P-PtdOH responses indicate that PLD is not involved (Figure 4A). Using a differential ^32^P_i_-labeling assay, ^32^P-PtdOH demonstrated to be rapidly labeled, in agreement with a DGK involvement, and in contrast to the labeling of structural phospholipids PtdEtn, PtdCho, and PtdGro, which required long labeling times, again suggesting a PLD-independent pathway (Figure 4B).

Although this seemed at variance with studies of suspension-cultured cells, which suggested a cold-activated PLD activity (Ruelland et al., 2002; Cantrel et al., 2011), it is well-possible that PLD plays a role at a later phase of the COR. This is for example supported by (1) the induced membrane localization of PLDδ after 1 day at 2°C (Kawamura and Uemura, 2003), (2) its importance in freezing tolerance (Li et al., 2004; Chen et al., 2008; Du et al., 2010) and (3) the transcriptional regulation of *Arabidopsis PLDδ* and *PLDα1* (Welti et al., 2002; Li et al., 2004) and two *PLDα* homologs from cotton (Kargiotidou et al., 2010) during cold acclimation. Nevertheless, consistent with the present data, we have found *pldα1*/*pldδ* seedlings to display a normal ^32^P-PtdOH response after 5 min at 0°C (Figure 4D).

### The substrate for DGK may be generated by a PtdInsP-hydrolyzing PLC

**Figure 8:**
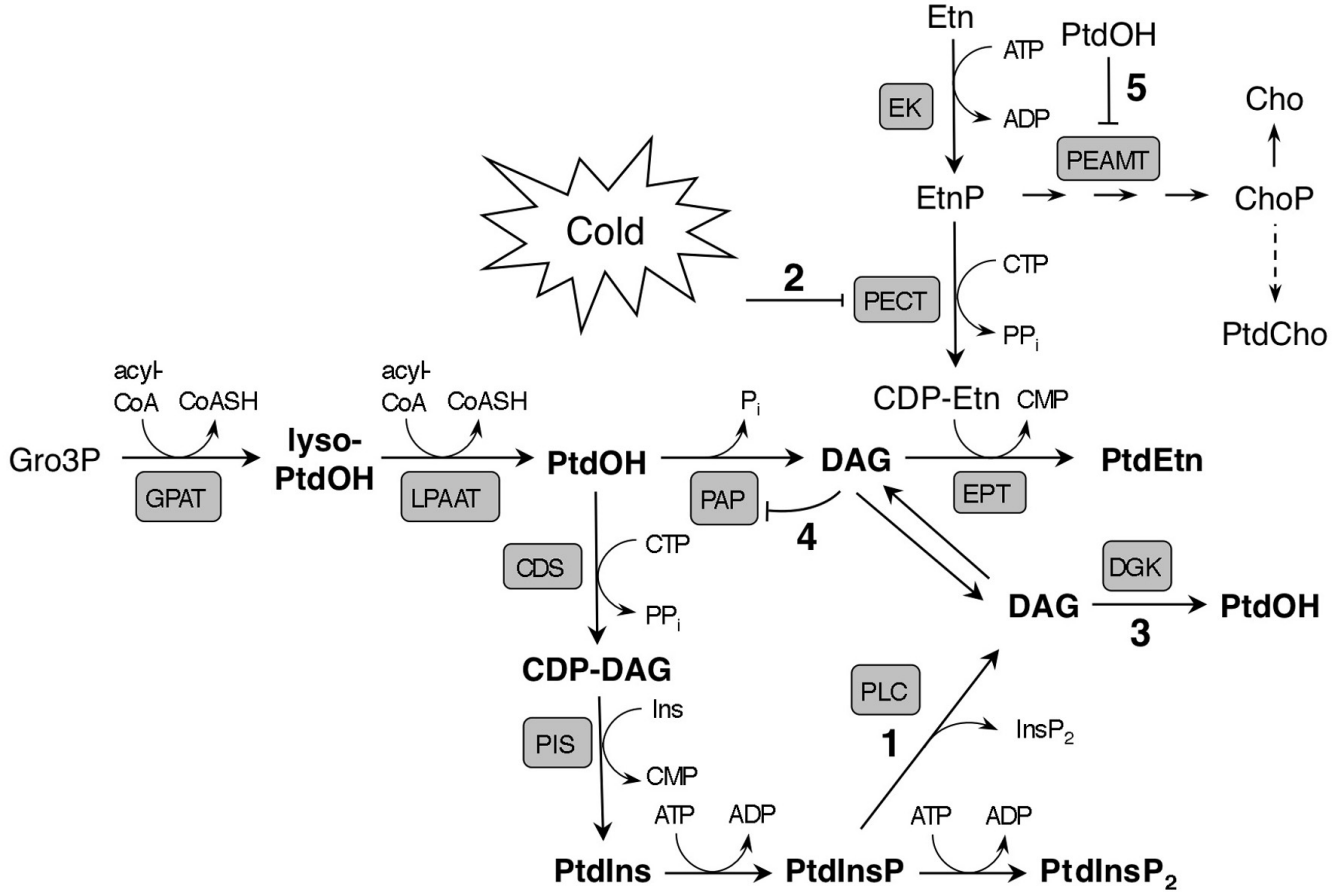
**Model illustrating potential early effects of cold stress on phospholipid metabolism and *de novo* synthesis in *Arabidopsis*.** The main route to rapid cold-induced PtdOH formation is suggested to be based on the phosphorylation of PLC-generated DAG from PtdInsP (reactions 1/3). The activity of PECT, which produces the precursor of the polar head of PtdEtn, CDP-Etn, is proposed to be down regulated by low ambient temperature (2). This would lead to reduced PtdEtn formation, and potentially, to DAG accumulation, which might cause PtdOH to accumulate as a result from phosphorylation of DAG by a DGK (3), or due to product inhibition of PAP by DAG (4). The major pathway of PtdCho synthesis depends on methylation of EtnP to ChoP by PEAMT, which could be inhibited by PtdOH (5). Note that the model only highlights immediate effects of cold temperature; longer exposure to cold induces a myriad of metabolic changes which impact lipid biosynthesis in different ways. *Abbreviations*: Acyl-CoA, acyl-coenzyme A; CDS, CDP-DAG synthase; EK, ethanolamine kinase; EPT, CDP-ethanolamine phosphotransferase; GPAT, glycerol 3-phosphate acyltransferase; LPAAT, lysophosphatidic acid acyltransferase; lyso-PtdOH, lysophosphatidic acid.

Since DGK was implicated, the question was raised how the substrate DAG was formed. Several pathways could account for this. A clue was provided by the concomitant decreases of ^32^P-PtdInsP, equivalent to the increase of ^32^P-PtdOH, suggesting the former to be precursor to DAG and PtdOH via PLC and DGK, respectively (Figures 5 and 8, reactions 1 and 3). Previously, cold stress in *Arabidopsis* cells has been shown to trigger decreases in both PtdInsP_2_ and PtdInsP (Ruelland et al., 2002). While PdInsP_2_ is usually considered as the substrate for PLC, in plants PtdInsP_2_ levels are extremely low, and, *in vitro*, PtdInsP is hydrolyzed equally well (Cho et al., 1993; Munnik et al., 1998a; Munnik and Testerink, 2009; Munnik and Vermeer, 2009).

### PtdOH response in T-DNA insertion lines

To answer the question which of the seven DGK isozyme(s) in *Arabidopsis* was responsible for the cold shock-induced PtdOH response, we tested T-DNA insertion lines with insertions in or near the coding regions, which caused suppressed transcript levels in some but not all (Figures A3 and A4). Nonetheless, neither seedlings (Table 1), nor leaf discs (Table 2) carrying the insertion mutations displayed an abrogation of the PtdOH response. In contrast, in both systems a knock-down allele of *DGK7, dgk7-2*, was associated with an increased accumulation of PtdOH. Knockdown of *DGK2*, did not result in significantly reduced PtdOH levels, perhaps as a consequence of the activity of the structurally similar *DGK1* (Gomez-Merino et al., 2004; Arisz et al., 2009).

These results may indicate a functional redundancy among *Arabidopsis* DGKs, such that deficient gene functions are compensated for by related isozymes. The PtdOH “overshoot” in the *dgk7-2* KD line could reflect such a mechanism. This experimental problem will be precluded by generating multigene KO lines, e.g., *dgk1*/*dgk2*.

In young seedlings, but also in rosette leaves, *DGK7* is more abundantly expressed than *DGK1* and *DGK2* (Arisz et al., 2009), but its transcript levels have not been found to increase upon cold stress. Transgenically expressed *DGK7* protein has been shown to have *in vitro* DGK activity despite the lack of a C1 domain, which is thought to function in the regulation of kinase activity in cluster-I DGKs, *DGK1* and *DGK2* (Gomez-Merino et al., 2004, 2005; Arisz et al., 2009). *DGK7* belongs to cluster II, together with *DGK3* and *DGK4* whose activity may be responsible for the enhanced PtdOH production in *dgk7-2* seedlings.

### Other potential sources of DAG and PtdOH

Although biochemical evidence strongly suggested a PLC-DGK route, two alternative enzymes might be considered as contributors to the cold-induced PtdOH accumulation as well.

**Figure A1:**
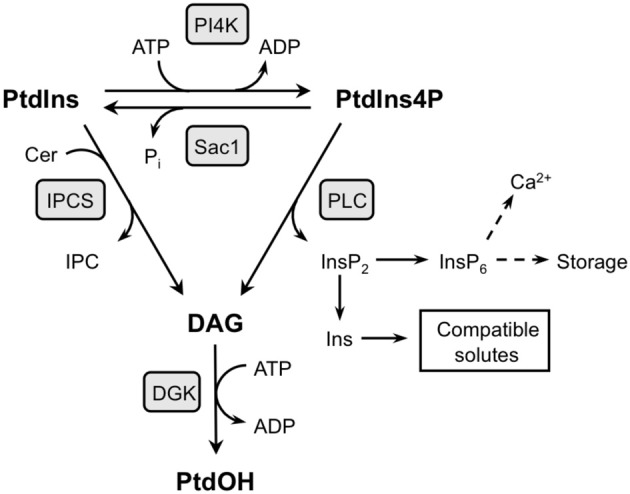
**Two pathways with the potential to generate DAG and PtdOH at the expense of PtdIns4P.** PtdIns4P is suggested to be the substrate of cold-induced PLC activity which not only generates DAG, but at the same time releases InsP_2_ that can be converted to InsP_6_ and/or Ins. The latter products may have a functional relevance in the stress response because InsP_6_ is a signaling compound in plants, and Ins is a precursor to compatible solutes (Vermeer and Munnik, 2010). Alternatively, inositolphosphorylceramide synthase (IPCS) generates DAG while transferring the InsP headgroup from PtdIns to ceramide (Cer), generating inositolphosphorylceramide (IPC). The PtdIns substrate in this conversion can be derived from PtdIns4P dephosphorylation, as, in yeast, through Sac1 activity. DAG generated via either of these pathways may subsequently be phosphorylated by DGK to generate PtdOH.

Inositolphosphorylceramide synthase (IPCS) transfers the inositol phosphate group from PtdIns to ceramide to generate inositolphosphorylceramide (IPC) and DAG (Figure A1). In yeast, the PtdIns substrate in this reaction is supplied through dephosphorylation of PtdIns4P by Sac1, coupling the consumption of PtdIns4P to the generation of DAG (Brice et al., 2009). An *Arabidopsis* IPCS, encoded by *ERH1*, has been implicated in pathogenic interactions (Wang et al., 2008). Interestingly, the physiological functions of PLC- and IPCS-mediated pathways may rely not only on the generation of inositol polyphosphates, IPC, and DAG/PtdOH, but also on the consumption of PtdInsP, which has novel functions in the biogenesis of secretory vesicles and the establishment/maintenance of cell polarity (reviewed by Munnik and Nielsen, 2011).

Recently, two interesting novel DAG sources have emerged in *Arabidopsis* stress responses: a PtdCho-hydrolyzing PLC, NPC4, that promotes tolerance to osmotic stresses (Peters et al., 2010), and SFR2, a galactolipid:galactolipid galactosyl transferase (GGGT), that produces DAG and oligogalactolipids to increase freezing tolerance (Thorlby et al., 2004; Moellering et al., 2010). For neither of the enzymes there is direct evidence that links their activity to stress-induced PtdOH accumulation. Rather, the enzymes could provide DAG as precursors for the synthesis of glycerolipids such as PtdCho and MGDG, or triacylglycerol, as for GGGT (Moellering et al., 2010; Moellering and Benning, 2011). It will nevertheless be interesting to subject KO mutants and overexpression lines of the corresponding genes to a differential PtdOH screen as we have applied in this study.

### PtdOH accumulation due to cold-induced inhibition of glycerolipid *de novo* synthesis?

**Figure A2:**
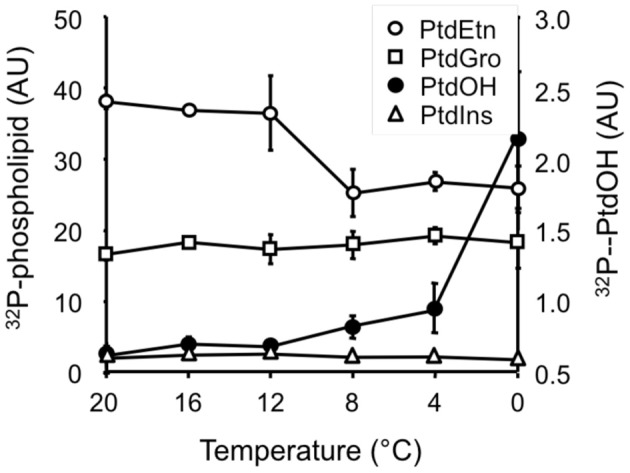
**Cold temperatures suppress ^32^P-PtdEtn accumulation in *Arabidopsis* leaf disks.** After 30 min ^32^P-labeling and 5 min incubation at the indicated temperatures, phospholipids were quantitatively analyzed by phosphoimaging. Values are in arbitrary units (AU) representing means (±SD) of the radioactivity levels.

**Figure A3:**
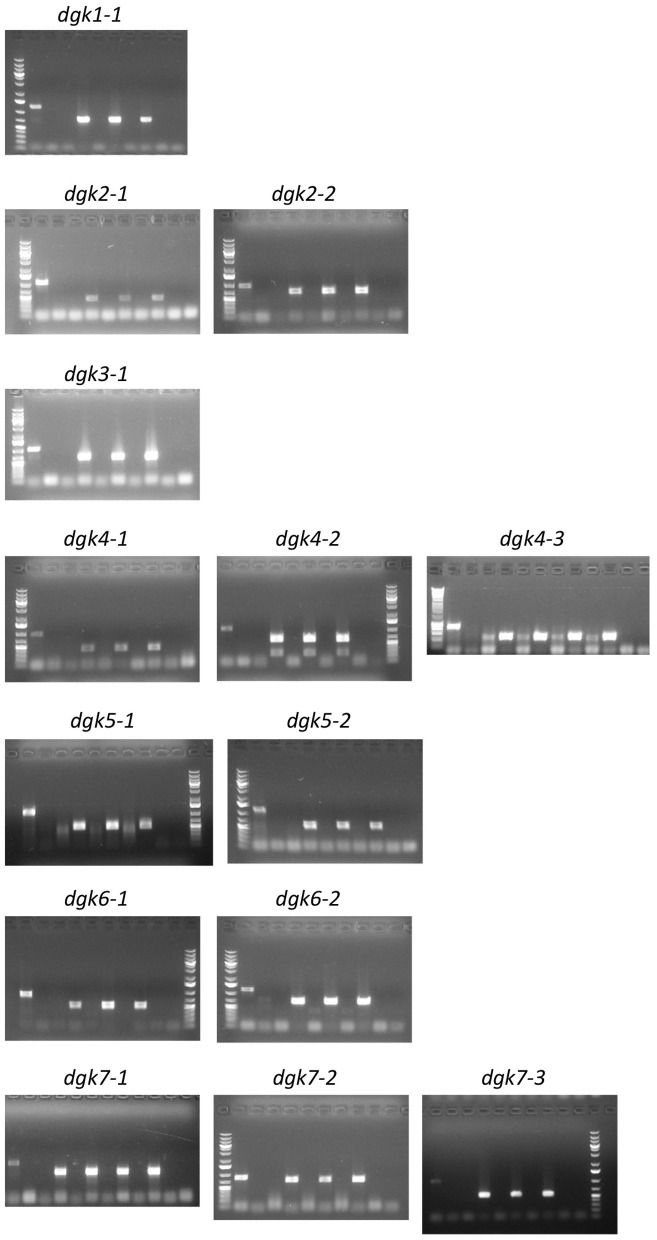
**Genotyping of the Arabidopsis *dgk* T-DNA insertion lines.** DNA was isolated from leaf discs from 3 to 4 independent plants and zygosity determined by PCR using specific wt- and mutant-primer sets as listed in Table A1. Gel sample order (from left to right): Size marker; Col-0 (wt), with first primer set, LP/RP (= wt band) and second primer set, RP/LB (= insertion band); Three (or four) independent dgk plants, with first primer set, LP/RP (= wt band) and second primer set RP/LB (= insertion band); Last lane, primer control (−). Some gels end with the size marker. Results clearly show that all lines used are homozygous T-DNA insertion lines since Col-0 was the only line giving wt bands and all insertion lines gave only bands with the second primer set. For the SALK lines LBb1.3 was used as LB primer, for the SAIL lines LB3 was used as LB primer (see primer list, Table A1).

**Figure A4:**
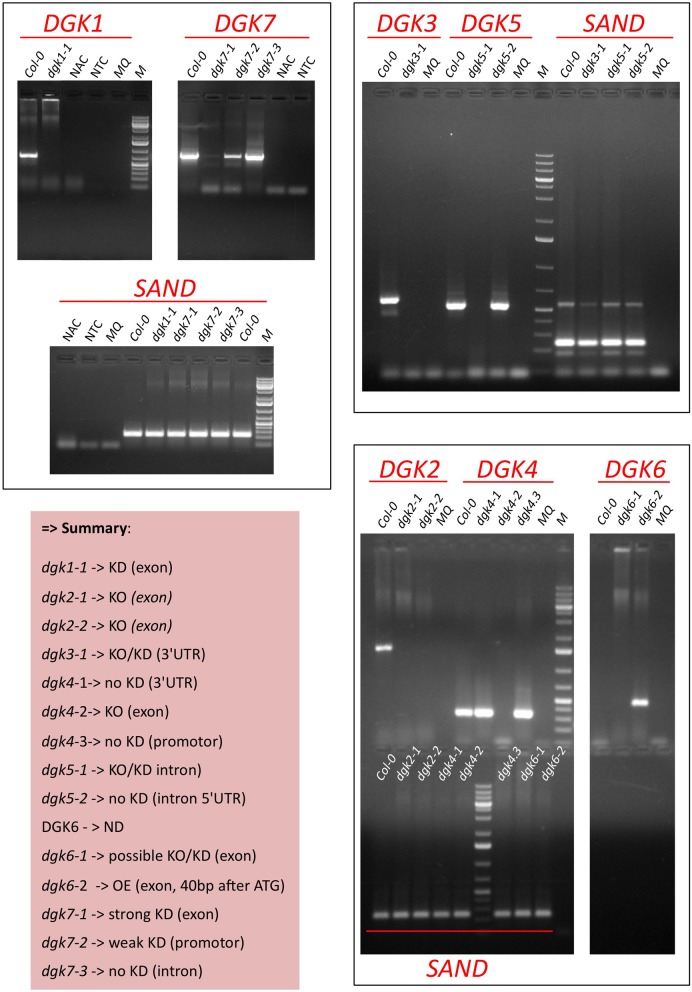
**Determination of *DGK* KO/KD-expression in Arabidopsis T-DNA insertion mutants by RT-PCR.** RNA was isolated from 9-days old roots *(DGK1,−3,−5,−7)* or flowers *(DGK2,−4,−6)* since the latter genes did not reveal expression in the root (not shown). Predicted band Sizes: *DGK1*, 702 bp; *DGK3*, 592 bp; *DGK5*, 534 bp; *DGK6*, 434 bp; *DGK7*, 791 bp; *SAND* (reference gene), 244 bp. *Abbreviations:* ND, not detectable; NAC, non-amplification control (test for genomic DNA contamination), i.e., RT reaction without RT enzyme on Col-0 RNA; NTC, no target control (test for contamination + primer dimers), i.e., RT reaction with water sample.

Cold may have a direct impact on glycerolipid *de novo* synthesis as it lowers diffusion rates and decreases the number of substrate molecules that have sufficient energy to allow enzymatic reactions (Mahan et al., 2004). In fact, in our differential labeling experiments we noticed a cold-induced decrease in the rate of PtdEtn labeling, only visible after approximately 20–30 min of labeling when its *de novo* synthesis in seedlings can just be witnessed (Figure 4B). Also in leaf discs, ^32^P-PtdEtn labeling was decreased upon cold incubation, suggesting a cold-induced inhibition of PE's *de novo* biosynthesis (Figure A2). This effect occurred at temperatures ≤8°C, while the decrease remained constant at lower temperatures down to 0°C. In contrast, maximum ^32^P-PtdOH accumulation was achieved at 0°C (Figure A2).

Glycerolipid *de novo* synthesis starts with two acylations of Gro3P to generate PtdOH (Figure 8). For the synthesis of PtdIns (and PtdGro), PtdOH is converted to cytidine diphosphate-diacylglycerol (CDP-DAG), the substrate for phosphatidylinositol synthase (PIS). Alternatively, PtdOH is dephosphorylated by phosphatidic acid phosphatase (PAP) to generate DAG as substrate in a reaction by which phosphoethanolamine (EtnP) is transferred from cytidine diphosphate-ethanolamine (CDP-Etn) to the lipid moiety, yielding PtdEtn. Cold does not seem to cause a general inhibition in the uptake of ^32^P_i_ or its incorporation into the Kennedy pathway of glycerolipid *de novo* synthesis, since labeling of other structural phospholipids was not affected by cold stress (Figure A2). Therefore, the cause of decreased ^32^P-PtdEtn labeling is most likely in the synthesis or supply of its headgroup.

The precursor CDP-Etn is generated through the cytidylation of EtnP by phosphoethanolamine cytidylyl transferase (PECT), analogous to the PtdCho headgroup precursor CDP-Cho, being the product of phosphocholine cytidylyl transferase (CCT) using ChoP as substrate. The latter is produced by repeated methylations of EtnP, catalyzed by phosphoethanolaminemethyltranferase (PEAMT). This activity, which is considered rate-limiting for PtdCho synthesis, likely accounts for the different labeling kinetics of PtdCho and PtdEtn, only the latter being radioactively detected after 30 min of ^32^P_i_-labeling.

As previous studies have shown that low temperatures can inhibit the *in vitro* activity of recombinant CCT (Inatsugi et al., 2002), PECT activity is speculated to be similarly downregulated by cold (Figure 8, designated by “2”), resulting in a limited availability of CDP-Etn for PtdEtn synthesis. This would form a bottleneck leading to the accumulation of DAG as precursor for PtdEtn synthesis. Notably, this DAG could be an additional source for cold-induced PtdOH through DGK activity (Figure 8, reaction 3), which has been shown to be partly localized at the ER in *Arabidopsis* (Vaultier et al., 2008). Alternatively, accumulated DAG may block its own formation through feedback inhibition of PtdOH phosphatase (Figure 8, reaction 4), again promoting PtdOH accumulation. Similar regulation of PAP activity by product inhibition has been demonstrated in chloroplast envelope membranes from spinach (Malherbe et al., 1992).

In summary, we have shown a very fast (in minutes) accumulation of PtdOH in response to cold temperatures in *Arabidopsis* seedlings and leaf discs, which was not due to PLD activity. Instead, ^32^P-radiolabeling studies indicated a dominant role of DGK under these conditions. Using single T-DNA insertion lines, we were unable to pinpoint the *DGK* gene(s) involved but do propose that DGK acts in tandem with a PtdInsP-hydrolyzing PLC, based on the close correlation between the increase in ^32^P-PtdOH and the decrease in ^32^P-PtdInsP.

PtdOH accumulation was not affected by the *fry1, hos1*, and *los1* mutations, consistent with an independent, upstream position in cold signaling. Although the *snow1* and *ice1* mutants displayed decreased PtdOH levels they likely reflected pleiotropic effects of the mutations.

Apart from the PLC/DGK route, additional, hypothetical sources of DAG and PtdOH were discussed, *viz*. via IPCS (Figure A1), NPC, GGGT and lipid *de novo* synthesis (Figure 8). Although for neither of these pathways there is sufficient evidence at present, they should not be ignored when studying PtdOH responses to cold or other environmental stresses.

### Conflict of interest statement

The authors declare that the research was conducted in the absence of any commercial or financial relationships that could be construed as a potential conflict of interest.

## Appendix

**Table A1:** **Primers used for genotyping the DGK T-DNA insertion lines**.

**Genotype**		**LP**	**RP**	**Border primer**	**Sequence**	**Wild type**	**Insertion**	**LP/RP**	**RP/LB**	**Insertion**
*dgk1-1*	SALK_053412	GGA TTC TCC TCC CGT AGA TTG	TCA TGC CGT ACT GGA AAA TTC	LBb1.3	ATTTTGCCGATTTCGGAAC	LP/RP	RP/LBb1.3	1205 bp	588−888 bp	chr5 2527025
*dgk2-1*	SAIL_718_G03	GCA AAG AAC AAA AAG GCA CAG	CAG ATG CAA GAC CGC TTT TAG	LB3	TAGCATCTGAATTTCATAAC CAATCTCGATACAC	LP/RP	RP/LB3	1106 bp	439−739 bp	chr5 25520634
*dgk2-2*	SAIL_71_B03	TTG TAAC TGG ATC TGT TGG CC	CTA AAA GCG GTC TTG CAT CTG	LB3	TAGCATCTGAATTTCATAAC CAATCTCGATACAC	LP/RP	RP/LB3	957 bp	432−732 bp	chr5 25520003
*dgk3-1*	SALK_082600	TGC TCT CAG TGG GAA GAG ATC	CCG GAA AAC TAT CCG GTT AAC	LBb1.3	ATTTTGCCGATTTCGGAAC	LP/RP	RP/LBb1.3	1179 bp	539−839 bp	chr2 8121795
*dgk4-1*	SAIL_339_C01	AAA GCG AAG CCG ATA TAA AGC	GTC TTT GGC AAA TCG TGG TAG	LB3	TAGCATCTGAATTTCATAAC CAATCTCGATACAC	LP/RP	RP/LB3	1011 bp	447−747 bp	chr5 23371938
*dgk4-2*	SALK_069158	TTT GAT CCC ATC GAA ACT CAC	GCC GAT GAT GGA CTA CTT GAG	LBb1.3	ATTTTGCCGATTTCGGAAC	LP/RP	RP/LBb1.3	1096 bp	448−748 bp	chr5 23372207
*dgk4-3*	SALK_151239	GGG AGC AGA ATC AGC AGG AAA	GAA TCA TCC TCG CCG TCA ATG	LBb1.3	ATTTTGCCGATTTCGGAAC	LP/RP	RP/LBb1.3	929 bp	?	chr5 23374706
*dgk5-1*	SAIL_1212_E10	TTC AGA GCA CAT GTG ACC AAC	TCC AAT TCG GAC ATT TGT TTC	LB3	TAGCATCTGAATTTCATAAC CAATCTCGATACAC	LP/RP	RP/LB3	1163 bp	505−805 bp	chr2 8990178
*dgk5-2*	SAIL_253_E12	GAC TTG AGC TGT TGC TGA TCC	GCG CAA CAA TTT TGG TAG AAG	LB3	TAGCATCTGAATTTCATAAC CAATCTCGATACAC	LP/RP	RP/LB3	1164 bp	536−836 bp	chr2 8993229
*dgk6-1*	SALK_016285	TGG GTA AAG TGA TCA ATG CAA AAG A	TGG CAA GCG AAA TTG GAA AGA	LBb1.3	ATTTTGCCGATTTCGGAAC	LP/RP	RP/LBb1.3	919 bp	?	chr4 13973176
*dgk6-2*	SALK_054320	GGG CCA TTA GTG GAA TTA AGC	CCT CCA GAT CAA AAA CCT GAG	LBb1.3	ATTTTGCCGATTTCGGAAC	LP/RP	RP/LBb1.3	1265 bp	606−906 bp	chr4 13971587
*dgk7-1*	SAIL_51_E04	TTT GCA AGA ATG CAT TTT TCC	TGC TGA TGG AGA TGT ACC TCC	LB3	TAGCATCTGAATTTCATAAC CAATCTCGATACAC	LP/RP	RP/LB3	1118 bp	434−734 bp	chr4 14839359
*dgk7-2*	SALK_059060	CAC GAT CTA ATA ACA CAC CAC ACC C	ACG ACC ACC ACT TTT CGG GTT	LBb1.3	ATTTTGCCGATTTCGGAAC	LP/RP	RP/LBb1.3	904 bp	?	chr4 14841224
*dgk7-3*	SALK_007896	CTC CAG GAG TTT TAG TTG GGG	CCG AAC ACG TTC TGT TAA AGC	LBb1.3	ATTTTGCCGATTTCGGAAC	LP/RP	RP/LBb1.3	1056 bp	457−757 bp	chr4 14839179

**Table A2:** **Primers used for RT-PCR expression analyses of DGK T-DNA insertion lines**.

**Gene**	**Direction**	**Primername**	**Sequence**
*DGK1*	sense	SALK_053412_LP	GGATTCTCCTCCCGTAGATTG
	antisense	SALK_053412_RP	TCATGCCGTACTGGAAAATTC
*DGK2*	sense	dgk2 fw	GACTGAGAGTTCCACTTTCTC
	antisense	dgk2 rv	GATCTACTCCACCCATATAGC
*DGK3*	sense	SALK_082600_LP (dgk3-1)	TGCTCTCAGTGGGAAGAGATC
	antisense	dgk3 rv	CAAACTTCATTCCTCACAACAC
*DGK4*	sense	dgk4 fw	GCAGTTGTTGCATTGAATCTAC
	antisense	dgk4 rv (A)	CCAAAGACTGGTGAGGGACTC
	antisense	dgk4 r3 (B)	CGCATCTTTCCAGTCTCCTC
*DGK5*	sense	dgk5 fw2	CCAGTGGCAGGACCTCCAC
	antisense	dgk5 rv	GGAATCTTGAAGGTATCCGCAG
*DGK6*	sense	dgk6 fw	CCTGGAACAGATAGGTCTTCG
	antisense	dgk6 rv	CATTGGCCATTTCTATCACAAATCTG
*DGK7*	sense	SALK_007896_LP	CTCCAGGAGTTTTAGTTGGGG
	antisense	dgk7 rv	GTTGTTTCCATGGTTCACCATCC
*SAND ref*	sense	At2g28390-Q-fw	CAGACAAGGCGATGGCGATA
	antisense	At2g28390-Q-rv	GCTTTCTCTCAAGGGTTTCTGGGT

Sequences of the different primer pairs used to measure the expression of the various DGK genes used in this study. SAND (At2g28390) expression was used as the reference gene (Hong et al., 2010).
